# Intervention Design With Cognitively Impaired Populations: The Optimize Deprescribing Intervention

**DOI:** 10.1093/geroni/igab046.1558

**Published:** 2021-12-17

**Authors:** Orla Sheehan, Elizabeth Bayliss, Ariel Green, Melanie Drace, Jonathan Norton, Emily Reeve, Kathy Gleason, Cynthia Boyd

**Affiliations:** 1 Johns Hopkins University School of Medicine, Johns Hopkins University, Maryland, United States; 2 Kaiser Permanente Colorado, Denver, Colorado, United States; 3 Johns Hopkins University School of Medicine, Baltimore, Maryland, United States; 4 Johns Hopkins University, Baltimore, Maryland, United States; 5 University of South Australia, Adelaide, South Australia, Australia; 6 Kaiser Permanente, Kaiser Permanente Institute for Health Research, Colorado, United States

## Abstract

Older adults with cognitive impairment and multiple other chronic conditions often have polypharmacy which increases their risks of medication related cognitive effects, adverse drug events, hospitalization and death and leads to higher health care costs. Deprescribing, the process of reducing or stopping potentially inappropriate medications may improve outcomes for those older adults with cognitive impairment and multiple chronic conditions. The OPTIMIZE trial examined whether a primary care-based, patient- and family-centered intervention educating and activating patients, family members, and clinicians about deprescribing reduces numbers of chronic medications and potentially inappropriate medications for older adults with dementia or mild cognitive impairment and multiple chronic conditions. We explored the mechanisms of intervention effectiveness through post hoc qualitative stakeholder interviews and surveys with 15 patients, 7 family caregivers, and 28 clinicians. All stakeholder groups endorsed the acceptability of the intervention. Success of the intervention was affected by contextual factors including prior knowledge and openness to deprescribing, cognition and prognosis. Positive outcomes included patients and care partners scheduling specific appointments to discuss deprescribing and providers remembering to consider deprescribing in cognitively impaired older adults. Recollection of intervention materials was inconsistent over time but highest shortly after intervention delivery. The time required to mail intervention materials to patients prior to a scheduled appointment limited the reach of the intervention by excluding persons with rapidly scheduled appointments. Our work identifies key learnings in intervention roll out which can guide future translation of our intervention to other settings and other pragmatic intervention studies in this vulnerable population.

